# Structural Changes Likely Cause Chemical Differences between Empty and Full AAV Capsids

**DOI:** 10.3390/biomedicines12092128

**Published:** 2024-09-19

**Authors:** Caryn L. Heldt, Molly A. Skinner, Ganesh S. Anand

**Affiliations:** 1 Department of Chemical Engineering, Michigan Technological University, Houghton, MI 49931, USA; mskinner@mtu.edu; 2 Health Research Institute, Michigan Technological University, Houghton, MI 49931, USA; 3 Department of Chemistry, Pennsylvania State University, University Park, PA 16802, USA; gsa5089@psu.edu

**Keywords:** gene therapy, cryo-EM, virus structure, biomanufacturing

## Abstract

Due to the success of adeno associated viruses (AAVs) in treating single-gene diseases, improved manufacturing technology is now needed to meet their demand. The largest challenge is creating a process to separate empty and full capsids. Patients received larger capsid doses than necessary due to the presence of empty capsids. By enabling the better separation of empty and full capsids, patients would receive the greatest therapeutic benefit with the least amount of virus capsids, thus limiting potential side effects from empty capsids. The two most common empty/full separation methods used in downstream processing are ultracentrifugation and anion exchange chromatography. Both processes have limitations, leading to a need for the identification of other structural differences that can be exploited to separate empty and full capsids. Here, we describe four possible theories of the structural changes that occur when AAV capsids envelop a genome. These theories include conformational changes occurring due to either the expansion or contraction of the capsid in the presence of nucleic acids, the constraining of the N-terminus into the five-fold pore when the genome is present, and the increased number of VP3 proteins in full capsids. These theories may reveal structural differences that can be exploited to separate full and empty capsids during manufacturing.

## 1. Introduction

Adeno-associated viruses (AAVs) are popular gene therapy vectors because of their large tissue tropism and low toxicity [1]. However, as the expansion of the applications for AAV gene therapy vectors continues, their high-dose applications are leading to toxic immunogenic side effects that include cytokine storms, progressive liver dysfunction, and death [2]. It was once thought that empty capsids could be a decoy for the immune system, thus making the immune system clear the empty capsids and allowing for the transgenic capsids to reach the target cell for transduction [3]. However, it has been shown that empty capsids may interfere with transduction, thus inducing cytotoxic responses [4,5] and/or clearance of transduced cells from the body [5]. This has led to a need, in their manufacturing, to separate empty capsids from full capsids to ensure that AAV gene therapies are providing the greatest therapeutic benefit with the least amount of virus capsids.

AAVs are non-enveloped viruses which encapsulate a 4.7 kb ssDNA genome. Each capsid is made up of 60 viral proteins consisting of varying ratios of VP1, VP2, and VP3, which are all encoded by the *cap* gene through alternative splicing and differing start codons. VP1 is the largest viral protein and all of VP2 is contained within VP1 and all of VP3 is contained within VP2. AAVs have an icosahedral symmetry and the most dominant feature of their structure is their 5-fold pore.

AAVs have a unique biology that leads to the generation of a large proportion of empty capsids during virus assembly. While many viruses condense capsid proteins around the nucleic acids they encapsulate, AAVs use *rep* proteins to insert their DNA into the 5-fold pore of a pre-formed capsid [6]. All four of the *rep* proteins come from the same open reading frame (ORF) and it is currently being explored which of these proteins are required for genome packaging [7,8,9]. This also increases the nonspecific packaging of DNA fragments, which are not useful for therapeutic applications [10]. Much work is being conducted to increase the production of full capsids; this includes engineering more efficient *rep* packaging proteins and increasing the egress of AAVs from the cell through the membrane-associated accessory protein (MAAP) [11]. However, until more progress is made in cell culture, the removal of empty capsids still lies solely within purification processes.

The two most common methods used to remove empty capsids in AAV manufacturing are gradient ultracentrifugation and anion exchange chromatography (AEX) [12,13]. Ultracentrifugation creates a very pure vector product, often with >80% full capsids, but it is not a sustainable strategy for producing vectors at scale. A more relevant separation method for large-scale manufacturing is anion exchange chromatography (AEX). AEX is reliably used for analytical purposes because the charge difference between empty and full capsids is small. For large, industrial-scale chromatography, AEX and multimodal chromatography (AEX and hydrophobic interactions) can be used to enrich full capsids, but the success of AEX is highly serotype-dependent [12,14,15]. Recently, the mechanistic modeling of the binding kinetics of empty and full capsids was used to determine ion exchange parameters that can separate AAV capsids [16]. A better understanding of the difference between empty and full capsids’ structural and binding characteristics could lead to standardization of methods for separating empty and full capsids.

Single particle Cryo-EM has struggled to capture the interior nucleic acid cores and changes in viral structure uponnucleic acid packaging. This is a consequence of the high dynamics of the nucleic acid genome within the virus particle. Cryo-EM approaches are an averaged snapshot of the end-state conformation of a whole viral particle in solution, without any insights into the conformational heterogeneity of the viral particles in solution. Every viral particle has a unique dynamic profile in solution that is a direct consequence of each virus having a distinct thermodynamic fingerprint that is related to the contacts between the capsid proteins, the packaging of genomic material, and other macromolecular interactions. Cryo-EM models are averaged over multiple particles, making it difficult to explore the heterogeneity of virus structures. It has been predicted that the ratio of VP1:VP2:VP3 varies widely in each virus preparation, with the largest composition being less than 2.5% of the population [17], thus demonstrating the heterogeneity that is found in AAVs. Other methods beyond cryo-EM are needed to explore the heterogeneity of viral particles and how the genome content affects their structure.

Virus structural assemblies are stochastic and also dynamic. Despite sharing similarities in their average structural organization, as evident from X-ray crystallography and cryo-EM, limited proteolysis mass spectrometry and, more recently, amide hydrogen/deuterium exchange mass spectrometry (HDXMS) have revealed that every viral particle is a spring-loaded dynamic entity that undergoes reversible conformational fluctuations, known as ‘breathing’ [18,19]. These intrinsic dynamics have been observed across multiple virus families [20]. A more complete readout of the virus’s structure, breathing, and conformational changes requires multiple analytical methods [21].

Changes in capsid structure result in charge and hydrophobicity shifts on the AAV’s capsid surface. However, these shifts are dependent on the serotype of the AAV. Single particle measurements using the atomic force microscopy (AFM) of AAV serotypes 2 and 8 (AAV2 and AAV8, respectively) show that AAV2’s empty capsids have a stronger adhesion to a positively charged surface than full capsids, whereas the opposite was shown with AAV8 capsids [22]. Hydrophobicity differences were also found between empty and full capsids for AAV2 and AAV8. This change can affect the way that capsids behave during manufacturing and could be utilized for empty/full separations.

The inclusion of multiple methods will ensure that the disadvantages of singular methods will be overcome by another method. multi-angle dynamic light scattering (MADLS) can be utilized to determine particle sizes down to the nanometer level [23]. This can show whether there are size differences in capsids when a genomic material is packaged. However, if the differences are small enough, they may not be picked up by MADLS. While Western blots cannot determine the size of a complex as large as viruses, Western blots can be used on dissociated capsids to determine the number of VP proteins in full versus empty capsids [24]. Other methods to determine how the surface VPs are exposed are necessary. It has been shown that VPs can overlap each other on the surface and that there could be differences in how they overlap each other between empty and full capsids [25]. Molecular dynamic (MD) simulations have been used to study how viruses infect cells [26]. MDs may also tell us more about the structural dynamics of viral particles. More information gathered by a variety of methods will lead to a more comprehensive understanding of the structural and chemical changes between full and empty AAV capsids.

Overall, there is no clear picture of how the presence of genomic material changes capsids’ structure. Understanding the structural changes between empty and full AAV capsids could be fundamental to creating methods to separate these capsids, as well as understanding places in which to engineer capsids for their greater separation by charge or hydrophobicity. In addition to understanding empty/full separations, a picture of how the genome affects capsid stability and chemistry could be used to potentially engineer cellular systems to produce a higher percentage of full capsids. Here, we describe four possible theories on how the presence of DNA could change the capsid structure enough to change the charge and/or hydrophobicity of the capsid surface.

## 2. Theories of Structural Changes in Genome-Containing AAV Capsids

### 2.1. Theory 1: The Pressure of the DNA inside the Capsid Causes Expansion and Induces Minor Conformational Changes on Its Surface

It has been demonstrated that increases in packaged genome size increase the internal pressure of some viral capsids [27]. This is especially relevant for AAVs, which do not condense around the genome but use the metabolic energy of the host–cell to insert DNA into a preassembled capsid [28,29]. This would support the idea that increases in genome size would necessitate an expansion of the capsid and a corresponding change in conformation. Fully packaged bacteriophage capsids are stiffer than empty capsids [30]. However, changes may be serotype-dependent. AAV2 was found to be quite stiff, regardless of whether the capsid was empty, full, or partially full [31]. There was an indication, however not a statistically significant one, that there may be a stiffness difference between empty and full capsids, but this needs further investigation. Packaging highly electronegative DNA in a tiny volume requires high metabolic energy, which could make the full AAV capsid a spring-loaded particle with high energy. This energy and dynamics could expand the AAV capsid and cause it to present different surface chemistries.

### 2.2. Theory 2: DNA in the Capsid Binds Tightly to the Positively Charged Amino Acids in the Internal Surface of the Capsid and Causes Some Amino Acids to Be Buried under the Surface

A second theory suggests that when the capsid and genome interact, the capsid slightly contracts, causing some of the surface amino acids to become buried under the surface [32]. Crystal structures show that the inner side of the AAV capsid is highly positively charged. This leads to the theory that the capsid highly interacts with the negatively charged DNA. This interaction could cause a contraction of the capsid, leading to changes in its surface chemistry.

Size changes have been observed for AAVs. Using multi-angle dynamic light scattering (MADLS), an empty AAV5 was found to be 6 nm (±0.7 nm) larger in diameter than a full capsid containing the GFP gene [23]. Cryo-EM analyses comparing empty and full AAV8 capsids showed that genome packaging elicited only small changes in average capsid assembly [32]. It found that three negatively charged amino acids had altered solvent accessibility, without changes in the hydrophobic amino acids [22]. This work supports the idea that the genome interacts with the interior of the viral capsid, causing contraction and charge differences between empty and full capsids.

### 2.3. Theory 3: The N-Tail of the Viral Capsid Protein Can Tuck into the Five-Fold Pore When DNA Is Present

In the five-fold pore, the N-tails of the VP are often unstructured [33]. Single-stranded RNA viruses are known to have the charged N-tails that tuck into the 5-fold pore to interact with the single-stranded RNA [34]. While AAVs are DNA viruses, they are also single-stranded. Single-stranded nucleic acids may increase the likelihood of the interaction of the unstructured N-tails with the nucleic acids, thus tucking the unstructured N-tail into the 5-fold pore. Cryo-EM shows that there is a lack of electron density in the N-terminal VP regions in purified AAV9 VLPs (empty capsids) [35]. This is an artifact of the icosahedral averaging carried out in cryo-EM analyses. The N-tail could either be located on the inside of the capsid [33], or it could support this theory that the N-tail is tucked into the pore when DNA is present [35]. However, there is evidence that N-tail tucking may not be the case for AAVs. The N-tail of the major VPs in AAVs are glycine-rich and contain negative charges [33]. However, without a clear indication of the N-tail structure, this is still a possible reason for the change in charge between empty and full AAV capsids.

### 2.4. Theory 4: Full Capsids Contain VP3 and Thus Have an Altered Capsid Structure

A recombinant AAV (rAAV) was found to incorporate a higher amount of VP3 in its full capsids compared to its empty ones [36]. This is the first demonstration that the assembly of the capsids could have an impact on the amount of empty or full capsids that are created. A lower amount of VP3 would change the capsid’s surface chemistry. However, AAV capsid assembly appears to be stochastic [17]. Therefore, the final ratio of capsid proteins in the final AAV structures is dynamic. But it is not clear whether the final ratio impacts the genome’s packaging. When speaking to AAV manufacturers, they have not seen a change in VP1, VP2, and VP3 ratios between empty and full capsids. This could be serotype-dependent, as we know that the ratio of VP1:VP2:VP3 varies between serotypes [17] and we need to continue to explore the correlation between the viral capsid proteins in empty and full capsids.

## 3. Concluding Remarks

While four different structural differences were described, evidence points to the most likely theory being the contraction/condensation of the capsids. This is the only theory that has been shown in multiple AAV serotypes as creating a possible difference between empty and full capsids. However, the field needs to continue to explore the structural differences between empty and full capsids for a variety of AAV serotypes, as the reason for a difference between empty and full capsids’ chemistry may be serotype-dependent.

While AEX is the most common separation method for empty and full AAV capsids, other methods could also be explored as we learn more about the structural differences between empty and full capsids. Hydrophobic differences could lead to the use of either hydrophobic interaction chromatography or, more likely, multimodal chromatography for the separation of empty and full capsids. Changes in shear stress and the deformation of empty capsids under high pressure could be used as a potential separation technique. Filtration using high pressure and precise pore sizes could squeeze empty capsids through pores, whereas the stiffer full capsids would stay in the retentate. The continued exploration of the structural differences between empty and full AAV capsids will allow for new and potentially less costly ways to remove empty capsids. This will lead to safer and less expensive gene therapy products and bring this miracle treatment to more people.

## Data Availability

No new data were created or analyzed as part of this study.
